# Betacoronavirus-specific alternate splicing

**DOI:** 10.1101/2021.07.02.450920

**Published:** 2021-07-02

**Authors:** Guy Karlebach, Bruce Aronow, Stephen B. Baylin, Daniel Butler, Jonathan Foox, Shawn Levy, Cem Meydan, Christopher Mozsary, Amanda M Saravia-Butler, Deanne M Taylor, Eve Wurtele, Christopher E Mason, Afshin Beheshti, Peter N Robinson

**Affiliations:** 1 The Jackson Laboratory for Genomic Medicine, Farmington, CT 06032, USA,; 2 COVID-19 International Research Team,; 3 Biomedical Informatics, Cincinnati Children’s Hospital Medical Center, Cincinnati, OH, 45229, USA,; 2,4 Department of Oncology, Sidney Kimmel Comprehensive Cancer Center, Johns Hopkins School of Medicine, Baltimore, MD 21287, USA,; 5 Department of Physiology and Biophysics, Weill Cornell Medicine, New York, NY USA,; 6 Logyx, LLC, Mountain View, CA 94043, USA,; 7 Space Biosciences Division, NASA Ames Research Center, Moffett Field, CA 94035, USA,; 8 Department of Biomedical and Health Informatics, The Children’s Hospital of Philadelphia, Philadelphia, PA 19104 USA,; 9 Department of Pediatrics, Perelman School of Medicine, University of Pennsylvania,Philadelphia, PA 19104 USA,; 10 Bioinformatics and Computational Biology Program, Iowa State University, Ames, IA, 50011, USA,; 11 Center for Metabolic Biology, Iowa State University, Ames, IA, 50011, USA,; 12 Genetics Development and Cell Biology, Iowa State University, Ames, IA, 50011, USA,; 13 Stanley Center for Psychiatric Research, Broad Institute of MIT and Harvard, Cambridge, MA, 02142, USA,; 14 KBR, Space Biosciences Division, NASA Ames Research Center, Moffett Field, CA, 94035, USA,; 15 Institute for Systems Genomics, University of Connecticut, Farmington, CT, USA,

## Abstract

Viruses can subvert a number of cellular processes in order to block innate antiviral responses, and many viruses interact with cellular splicing machinery. SARS-CoV-2 infection was shown to suppress global mRNA splicing, and at least 10 SARS-CoV-2 proteins bind specifically to one or more human RNAs. Here, we investigate 17 published experimental and clinical datasets related to SARS-CoV-2 infection as well as datasets from the betacoronaviruses SARS-CoV and MERS as well as Streptococcus pneumonia, HCV, Zika virus, Dengue virus, influenza H3N2, and RSV. We show that genes showing differential alternative splicing in SARS-CoV-2 have a similar functional profile to those of SARS-CoV and MERS and affect a diverse set of genes and biological functions, including many closely related to virus biology. Additionally, the differentially spliced transcripts of cells infected by coronaviruses were more likely to undergo intron-retention, contain a pseudouridine modification and a smaller number of exons than differentially spliced transcripts in the control groups. Viral load in clinical COVID-19 samples was correlated with isoform distribution of differentially spliced genes. A significantly higher number of ribosomal genes are affected by DAS and DGE in betacoronavirus samples, and the betacoronavirus differentially spliced genes are depleted for binding sites of RNA-binding proteins. Our results demonstrate characteristic patterns of differential splicing in cells infected by SARS-CoV-2, SARS-CoV, and MERS, potentially modifying a broad range of cellular functions and affecting a diverse set of genes and biological functions.

## Introduction

Coronavirus SARS-CoV-2 emerged in late 2019 as the third human coronavirus identified in the 21st century. Coronavirus disease 2019 (COVID-19) affects diverse organ systems, including the lungs, digestive tract, kidneys, heart, and brain and is associated with extremely heterogeneous manifestations that range from minimal symptoms to significant hypoxia with acute respiratory distress requiring mechanical ventilation [1, 2]. Coronaviruses are large, enveloped, single-stranded RNA viruses found in humans and other mammals and can cause respiratory, gastrointestinal, and neurological disease. In addition to SARSCoV-2, two other betacoronaviruses associated with severe disease led to global outbreaks in the last two decades: the Severe Acute Respiratory Syndrome (SARS)-associated coronavirus (SARS-CoV) first reported in 2003 and the Middle East Respiratory Syndrome (MERS)-associated coronavirus (MERS-CoV) first reported in 2012 [3].

Viral genomes encode a limited set of genes, and so viruses rely on the host cellular machinery. Viral components can subvert a number of cellular processes in ways that have evolved to block innate antiviral responses. A number of viruses have been shown to interact with cellular splicing machinery. The process of precursor mRNA (pre-mRNA) splicing involves the removal of introns and the precise joining of exons to form mature mRNA molecules. Over 95% of human genes undergo alternative splicing in a developmental, tissue-specific, or signal transduction-dependent manner. Alternative splicing plays important roles in development, disease, and aging [4]. Although some splicing isoforms are produced in the same proportions in all or most cell types, alternative splicing is often regulated by developmental or differential cues or in response to external stimuli [5]. Modulation of splicing has been shown to be an important mechanism in the host’s response to viral infection [6, 7, 8]. On the other hand, viruses can alter splicing patterns. For instance, the Dengue virus NS5 protein binds to core components of the U5 snRNP, and incorporates in active spliceosomes and pre-mRNA processing. Dengue-virus induced changes in the isoform abundance of antiviral factors IKBKE (inhibitor of *k*B kinase *ϵ*) in a way that could facilitate viral replication [9]. *MX1* encodes an antiviral protein that is induced by interferon-*α*/*β* and inhibits the replication of many RNA viruses. Both Dengue virus and Herpes simplex virus −1 (HSV1) induces alternative splicing in *MX1* that supports instead of restricting viral infection [10, 11, 9]. Poliovirus infection can result in cleavage of a component of the macromolecular SMN complex that mediates assembly of U snRNP complexes by aiding the heptameric oligomerization of Sm proteins onto U snRNAs [12]. Influenza A encodes a protein that modulates mRNA splicing to degrade the mRNA that encodes RIG-I, which encodes a protein that detect the presence of viral RNAs [13].

At least 10 SARS-CoV-2 proteins show specific binding to one or more human RNAs, including 6 structural non-coding RNAs and 142 mRNAs. A highly specific interaction was shown between the SARS-CoV-2 NSP16 protein and human U1 and U2 snRNAs, which hybridize to the 5’ splice site and to the branchpoint, respectively [14]. NSP16 is an RNA cap modifying enzyme with methytransferase activity that modifies viral RNAs [15]. It was shown that NSP16 additionally suppresses host mRNA splicing. SARS-CoV-2 infection and transfection with NSP16 result in an increase in intron retention in multiple IFN-responsive genes, thereby suggesting a role in splicing in suppressing the host interferon response to SARS-CoV-2 infection [14]. Another non-structural protein, NSP1, whose homologs in SARS-CoV and MERS-CoV have roles in viral replication, translational inhibition, transcriptional inhibition, mRNA degradation, and cell cycle arrest, was shown to contribute to global inhibition of host protein translation and manipulating translation [16, 17, 18]; in contrast, the translation of SARS-CoV-2-encoded subgenomic RNAs, which contain a common 5’ leader sequence that is added during negative-strand synthesis is not suppressed. NSP8 and NSP9 bind to the 7SL RNA component of the signal recognition particle (SRP) and interfere with protein trafficking to the cell membrane upon infection. NSP8 and NSP9 appear to be involved in suppression of the interferon response, which is dependent upon the SRP pathway for transport [14].

Here, we present a comprehensive survey of alternative splicing associated with infection by SARS-CoV-2, the two other betacoronaviruses SARS-CoV and MERS, and six other viruses and *Streptococcus pneumonia*, and show associations of betacoronavirus infection with a number of cellular parameters, affecting genes involved in a wide range of biological functions.

## Methods

### RNA-seq data sources

We investigated a range of RNA-seq datasets in which primary cells or cell lines were infected with different pathogens including the three betacoronaviruses as well as the influenza virus H3N2, the bacterium *Streptococcus pneumonia*, Hepatitis C virus, Zika virus, Dengue virus, and Respiratory Syncytial Virus. All these datasets were downloaded from NCBI’s Short Read Archive (SRA) [19]. Infection of cell lines or primary cells with virus is a commonly used experimental system to investigate host dependency factors and host restriction factors [20].

An additional, clinical dataset was analyzed from nasopharyngeal swab specimens (NPSS) from the New York-Presbyterian Hospital-Weill Cornell Medical Center [27]. We will refer to this dataset as SARS-CoV-2-A in the text. Briefly, nasopharyngeal swabs were collected using the BD Universal Viral Transport Media system (Becton, Dickinson and Company, Franklin Lakes, NJ) from symptomatic patients. Total Nucleic Acid (TNA) was extracted using automated nucleic acid extraction on the QIAsymphony and the DSP Virus/Pathogen Mini Kit (Qiagen). RNA isolation and library preparation is fully described in Butler, et al. [27].

### Mapping of RNA-seq data and isoform calling

For each cohort listed in Table 1, RNA-seq data were obtained from the NCBI SRA resource [19]. Except for the nasal swab (NSPP) dataset [27], all datasets were processed using a snakemake pipeline that performs the following steps: samples are downloaded from the SRA, quality-control is performed using fastp [30], alignment to Genome Reference Consortium Human Build 38 version 91 is done using STAR [31], and isoform quantification carried out by RSEM [32].

Raw RNA sequence data from the nasopharyngeal swab samples (SARS-CoV-2-A) were generated as described [27]. Reads classified as human using Kraken2 [33] were processed as described in https://github.com/asaravia-butler/COV-IRT/blob/main/RNAseq/Raw_to_Aligned_Data_Pipeline.md and https://github.com/asaravia-butler/COV-IRT/blob/main/RNAseq/RSEM_Counts_Pipeline.md. First, adapters and low-quality data were trimmed with Trimmomatic (v0.39) [34]. Kraken2-human classified raw and trimmed read quality were evaluated with FastQC (v0.11.9) (https://www.bioinformatics.babraham.ac.uk/projects/fastqc/), and MultiQC (v1.9) [35] was used to generate MultiQC reports. Trimmed reads were split according to sequencing flow cell and lane using gdc-fastq-splitter (v1.0.0) (https://github.com/kmhernan/gdcfastq-splitter) for subsequent batch effect evaluation. *Homo sapiens* STAR and RSEM references were built using STAR (v2.7.3a) [31] and RSEM (v1.3.1) [32], respectively, with Ensembl release 100 human genome version GRCh38 (Homo_sapiens.GRCh38.dna.primary_assem concatenated with the SARS-CoV-2 Wuhan-Hu-1 SARS-CoV-2 reference genome ASM985889v3, and the following Ensembl gtf annotation file: Homo_sapiens.GRCh38.100.gtf concatenated with Sars_cov_2.ASM985889v3.101.gtf. rRNA-depleted trimmed reads were aligned to the Homo sapiens and SARS-CoV-2 STAR reference with STAR twopassMode (v2.7.3a) [31]. Transcriptome-aligned reads were quantified using RSEM (v1.3.1) [32] to generate isoform count data.

### Analysis of differential gene expression and splicing

We used HBA-DEALS [36] to calculate the probabilities of differential expression and differential splicing. HBA-DEALS automatically determines the hierarchy of the covariates *α* and *β* - they are either in the same model layer and predict isoform expression, or *β* is in a separate layer that predicts gene expression separately. The choice is made by computing a Bayes factor that compares the two models in a sample of genes, and selecting the model which the majority of Bayes factors favor. For accounting for multiple comparisons we use an Bayesian approach [37]. The prior for *α* and *β* is a mixture of two Dirichlet or two Gaussian components, respectively. The first component has a very large variance and it corresponds to differentially expressed or differentially spliced genes, where little is known about the effect size a-priori. The second component has a very small variance and is centered around 0 for *β* and around ($\frac{1}{T},\;\frac{1}{T},\;..,\frac{1}{T}$) for *α*, where *T* is the number of isoforms. This component corresponds to very small effects that are not biologically meaningful. Since the mixture probabilities are not known in advance, for example we do not know the number of differentially expressed genes, HBA-DEALS infers them from the data. It creates a model that includes all the genes and isoforms in the experiment, and finds the mixture probabilities at the mode of the posterior. The tool stan provides an L-BFGS optimizer which can be accessed via the ‘optimizing’ function in its R interface and which HBA-DEALS uses for this purpose. Once the mixture probabilities for the prior have been obtained, they are set as constant in the prior of each gene, and the full posterior is computed for each gene separately using MCMC sampling, which is executed via the ‘sampling’ function in stan’s R interface. In order to summarize the posteriors of *α* and *β* of each gene into probabilities of differential splicing and differential expression, respectively, HBA-DEALS sums the density of the posterior that falls within a region of parameter values that corresponds to the first component of the prior, i.e. the component that corresponds to differential expression or splicing. The region of parameter values that agrees with the second component of the prior is known as the Region of Practical Equivalence (ROPE) [38]. We place changes of 0.1 in log-expression and 0.2 or less in isoform proportion within the ROPE. After the individual probabilities of effect are calculated, the FDR is estimated as the mean of the probabilities of no effect. In this work we set an FDR threshold of 0.05, and exclude any genes or isoforms with a probability of no-effect greater than 0.25 from the sets of differential genes or isoforms. HBA-DEALS is freely available at https://www.github.com/TheJacksonLaboratory/HBA-DEALS.

### Gene Ontology Analysis

We implemented a version of the Ontologizer [39] analysis code in our Java library phenol (https://github.com/monarch-initiative/phenol). We used the term-for-term GO enrichment analysis with Benjamini-Hochberg correction to select enriched GO terms with FDR≤0.05

### Calculating the Proportion of Retained Intron Isoforms

We used the Ensembl transcript IDs of isoforms included in the study to retrieve the transcript biotype field from Biomart [40] for each differentially spliced isoform. The proportion of retained intron isoforms is then the number of ‘retained intron’ biotypes divided by the total number of biotypes.

### Calculating the Mean Number of Exons of Isoforms

For each transcript, the number of exons in the GTF file Homo sapiens.GRCh38.91.gtf were counted, and the mean number of exons of differentially spliced isoforms was calculated for each dataset. The SARS-COV-2 nasal swab (NSPP) dataset was aligned to the genome using Homo sapiens.GRCh38.100.gtf, and therefore for this dataset we used this GTF file for counting the number of exons of each isoform.

## Results

In this study, we have performed an in-depth analysis using publicly available RNA-seq data representing experimental and clinical samples of human cells or tissues infected by a range of viruses and bacteria in order to characterize the biological processes that are affected by changes in gene expression and alternative splicing during SARS-CoV-2, SARS-CoV, and MERS infection and compared them to changes during infection by other viruses and bacteria.

### Disease-associated betacoronaviruses display characteristic functional profile of alternatively spliced genes

**Figure 1:**
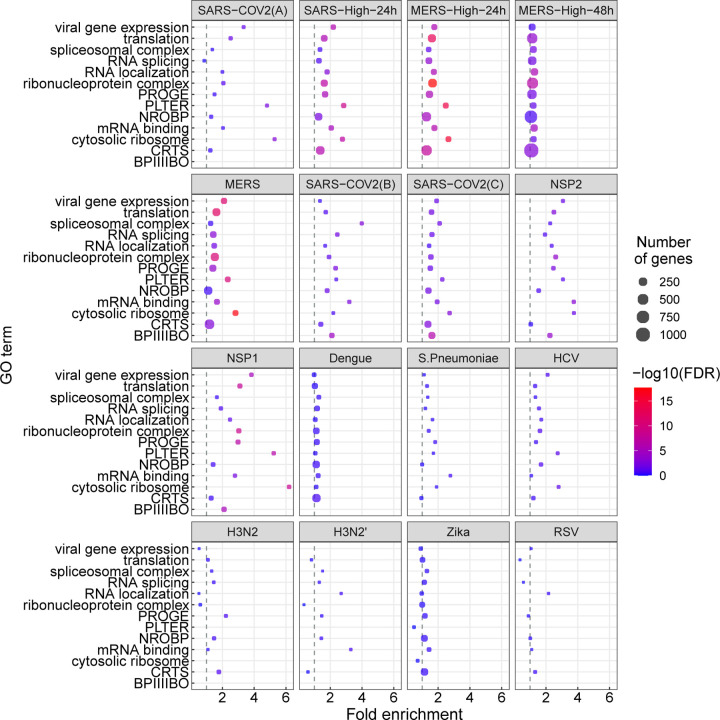
GO enrichment for genes showing differential alternative splicing (DAS). 14 representative GO terms were chosen from a total of 1,044 enriched terms (Supplemental Figure S1). The x-axis coordinate corresponds to the fold-change of the size of the GO term in the set of differentially-spliced genes compared to all the genes in the study. The size of the circle corresponds to the number of differentially-spliced genes that belong to the GO term. The color of the circle corresponds to the value of the −log_10_ of the corrected GO term enrichment p-values. Abbreviations. BPIIIIBO: biological process involved in interspecies interaction between organisms; CRTS: cellular response to stress; NROBP: negative regulation of biosynthetic process; PLTER: protein localization to endoplasmic reticulum; PROGE: posttranscriptional regulation of gene expression.

**Figure 2:**
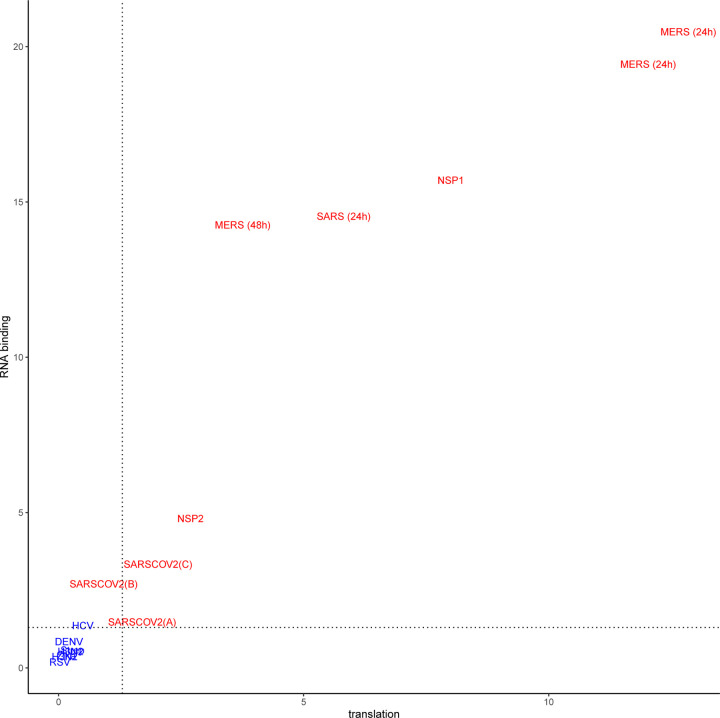
− log_10_(adjusted p value) for DAS enrichment for the GO terms *translation* (GO:0006412) and *RNA binding* (GO:0003723) in each of the datasets analyzed in this work. Betacoronavirus datasets are shown in red, others are shown in blue. The dashed lines correspond to an adjusted p-value of 0.05. Betacoronaviruses have higher enrichment scores for translation and RNA binding, suggesting that differential splicing has a larger impact on these processes. For abbreviations see Table 1.

In this study, we analyzed RNA-seq datasets representing experimental or clinical studies of SARS-CoV-2, SARS, MERS, four other viruses, and *Streptococcus pneumonia* (Table 1). We reasoned that although differences in experimental design and sample preparation preclude comparisons between individual datasets, a global analysis of profiles of genes and isoforms in each dataset could be used to generate hypotheses about characteristic functional profiles induced by alternative splicing in samples infected with viruses. We therefore performed Gene Ontology (GO) term enrichment analysis on each dataset and created a heat plot for all GO terms in which the BH-corrected *p*-value was less than 0.05 in at least two datasets; a total of 1,044 such terms were identified (Supplemental Figures S1–S2). Out of 1,044 terms that were enriched in at least two datasets, 1,025 terms showed a high degree of enrichment in at least one betacoronavirus dataset with respect to DAST. 130 of these terms had a median score below threshold (Supplemental Figure S3). For display purposes, we chose 14 representative GO terms (Figure 1). Figure 2 displays the −log10 of the adjusted p-value of DAS enrichment in each dataset for two GO terms: *translation* and *RNA binding*.

SARS-CoV-2 infection is reported to disrupt both translation and RNA splicing [14]. We therefore asked if the functional profile of DAS genes is enriched for Gene Ontology (GO) terms related to translation and RNA binding. We found that SARS-CoV-2, NSP1, NSP2, SARS, and MERS datasets showed enrichment for both *translation* (GO:0006412) and *RNA binding* (GO:0003723) and that the degree of enrichment was correlated (Figure 2).

**Figure 3:**
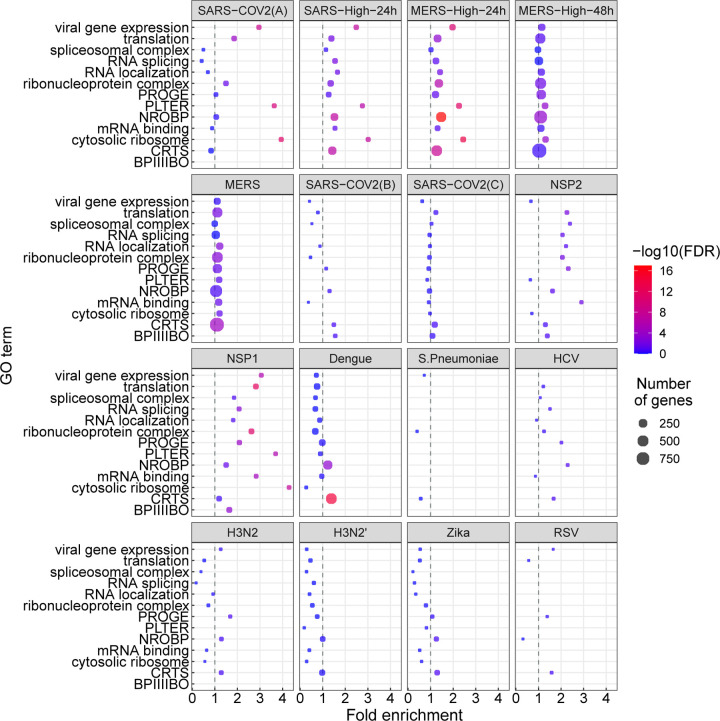
GO enrichment for genes showing differential gene expression (DGE). The GO terms and abbreviations are the same as in Figure 1. The x-axis coordinate corresponds to the fold-change of the size of the GO term in the set of differentially-expressed genes compared to all the genes in the study. The size of the circle corresponds to the number of differentially-expressed genes that belong to the GO term. The color of the circle corresponds to the value of the − log_10_ of the corrected GO term enrichment p-values.

We performed a similar analysis for differentially expressed genes (Figure 3). The terms showing enrichment in multiple betacoronavirus samples included terms directly related to viral infection including *viral gene expression*, as well as biological processes known to be involved in the cellular response to selected viral infections, including *mRNA splicing* and *spliceosomal complex* [41, 42, 43], *protein localization to endoplasmic reticulum* with a possible relation to endoplasmic reticulum stress [44], *cytosolic ribosome* [45], and *translation* [46]. Several of these GO terms were also enriched for differentially expressed genes (Figure 3).

### betacoronavirus samples display more intron retention

**Figure 4:**
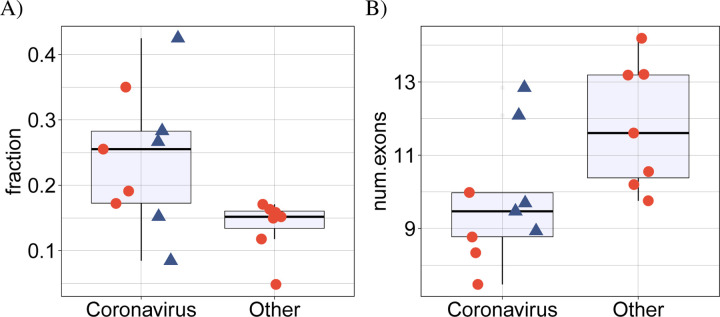
(A) Comparison of the mean proportion of intron retention isoforms in 9 coronavirus samples against the remaining 7 samples for the other pathogens in cyan. *p* = 0.016, Mann-Whitney-test. For each dataset, the mean proportion is calculated as the number of DAS isoforms annotated as retained intron isoforms is divided by the total number of DAS isoforms. (B) Comparison of the mean number of exons in 9 coronavirus samples against the remaining 7 samples for the other pathogens in cyan. *p* = 0.016, Mann-Whitney-test. For each dataset, the mean number of exons is calculated over all DAS isoforms. In both panels, the four SARS-CoV-2 datasets (SARS-CoV-2-A, SARS-CoV-2-B, SARS-CoV-2-C,NSP1 and NSP2) are shown as rectangles.

SARS-CoV-2 infection was previously shown to disrupt suppress global mRNA splicing [14]. We therefore hypothesized that infection with any of the betacoronaviruses could induce a greater degree of intron retention. We analyzed the mean proportion of intron-retention isoforms among all genes showing DAS and showed a significantly higher degree of intron retention in the betacoronavirus samples (Fig 4A). Additionally, the mean number of exons in isoforms that were significantly differentially spliced in the betacoronaviruses was lower (Fig 4B).

### More ribosomal genes are affected by DAS and DGE in betacoronavirus samples

**Figure 5:**
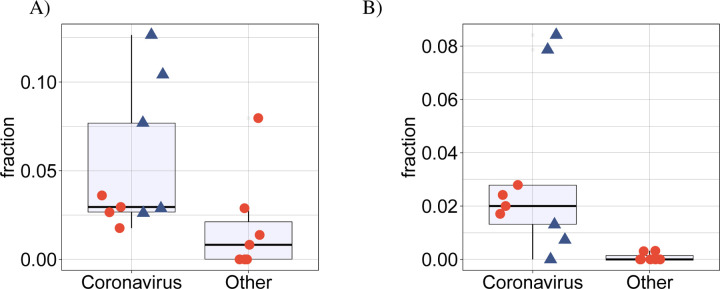
(A) Comparison of the mean proportion of DAS ribosomal genes in 7 coronavirus samples and cells infected with NSP1 or NSP2 against 7 samples infected with other pathogens. *p* = 0.033, Mann-Whitney-test. For each dataset, the mean proportion is calculated as the number of ribosomal genes containing DAS isoforms divided by the total number of genes containing DAS isoforms. (B) Comparison of the mean proportion of differentially expressed ribosomal genes in 7 coronavirus samples and cells infected with NSP1 or NSP2 against 7 samples infected with other pathogens. *p* = 0.004, Mann-Whitney-test. For each dataset, the mean proportion is calculated as the number of ribosomal genes that were differentially expressed divided by the total number of differentially expressed genes. In both panels, the four SARS-CoV-2 datasets (SARS-CoV-2-A, SARS-CoV-2-B, SARS-CoV-2-C, NSP1 and NSP2) are shown as rectangles.

Various ribosomal proteins (RPs) have been shown to participate in viral protein biosynthesis and regulate the replication and infection of virus in host cells [45]. We investigated whether the proportion of ribosomal genes displaying DAST and DGE differed between the betacoronavirus and other datasets. We found a significantly higher proportion of ribosomal genes that displayed DAST in the betacoronavirus samples (Fig. 5A).

A similar result was obtained for the proportion of ribosomal genes that were differentially expressed (*p* = 0.004, Mann-Whitney-test, Fig. 5B). It has been previously reported that the SARS-CoV-2 NSP1 protein can interfere with translation [47, 14]. Our findings support the conclusion that betacoronavirus infection involves or results in regulatory changes in the transcription of ribosomal genes.

### More betacoronavirus differentially spliced genes are affected by a pseudouridine modification

**Figure 6:**
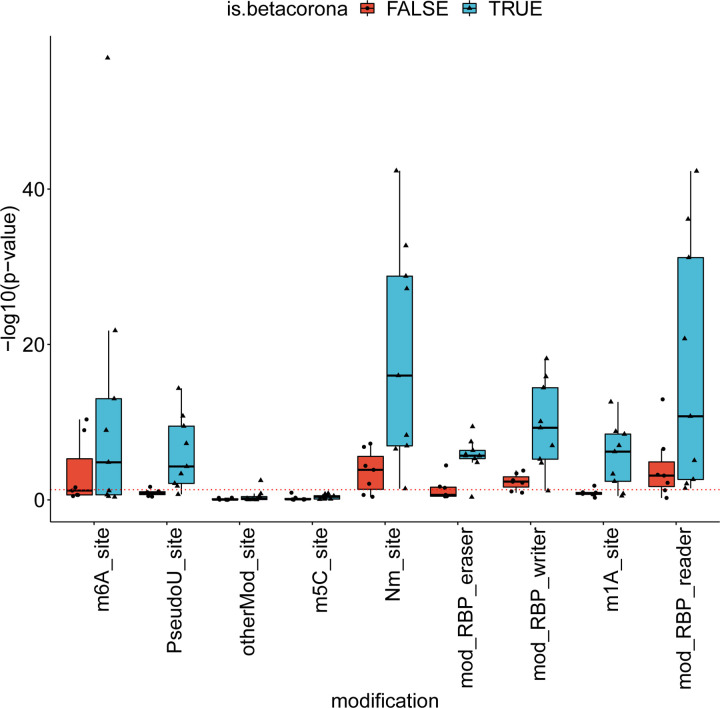
RNA modifications associated with betacoronavirus-enriched alternative splicing. The y-axis corresponds to the enrichment score of the modification in the set of differentially-spliced genes, calculated as − log_10_ of the hypergeometric test p-value. The dashed red line corresponds to a p-value of 0.05. RNA modifications were obtained from the RBM database ( [49]). Abbreviations: m6A: N6-methyladenine, PseudoU: pseudouridine, otherMod: other modification, m5c: 5-methylcytosine, Nm: 2^*′*^-O-methylation, RBP eraser: “erasers” of RNA modifications, RBP writer: “writers” of RNA modifications, m1a: N1-methyladenosine, RBP reader: “readers” of RNA modifications.

Pseudouridine is an RNA modification that has been shown to affect splicing [48]. Using the RBM database of RNA modifications [49], we calculated enrichment of RNA modifications in the datasets of this study and compared enrichment in DAS genes of betacoronaviruses and other pathogens. Figure 6 displays the enrichment score for the different datasets and different modifications, calculated as −*log*_10_(*p*), where *p* is the p-value is obtained using the hypergeometric test. Among the different modifications, Pseudouridine was the modification for which there was the largest difference between the number of betacoronavirus datasets that passed a significance threshold of p-value 0.05 and the number of other pathogen datasets that passed this threshold (Mann-Whitney test p-value 0.003, Fig. 6). This suggests that pseudouridine may be associated with some of the changes in alternative splicing induced during betacoronavirus infection. Other modifications were either enriched in smaller subsets of the betacoronavirus datasets or enriched in both betacoronaviruses and other datasets, suggesting that the modifications may be related to alternative splicing in general or alternative splicing that is triggered by an immune response.

### Differentially spliced genes of betacoronaviruses are depleted of RBP binding sites

**Figure 7:**
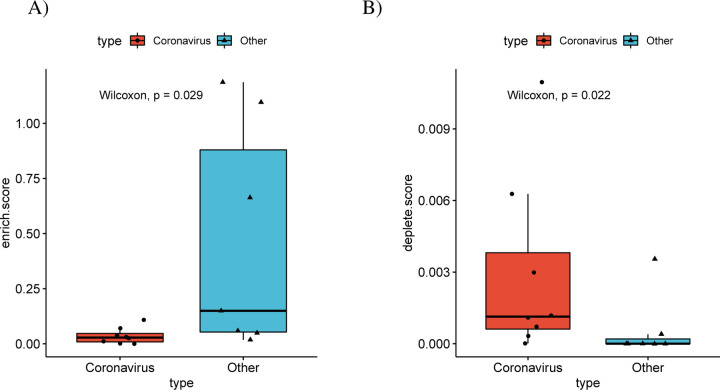
Differential splicing and enrichment/depletion of RNA Binding Protein Binding Sites. (A) For each dataset the number of RBP binding sites with enriched targets (adjusted p≤0.05,hypergeometric test) divided by the number of differentially spliced isoforms are displayed, with separate boxes for betacoronavirus datasets and other datasets(*p* = 0.0289, Mann-Whitney test). RBP binding sites were obtained from the ORNAment database [50](B) Boxplots of the number of RBP binding sites with depleted targets (adjusted p≤0.05,hypergeometric test) divided by the number of non-differentially spliced isoforms (*p* = 0.0216, Mann-Whitney test).

In order to investigate the mechanisms that determine the observed alternative splicing patterns, we calculated the number of binding sites of RNA Binding Proteins (RBPs) in the sets of differentially spliced genes. For this purpose, we downloaded all RBP targets in the human genome from oRNAment [50]. For each RBP binding site, we determined the p-value of observing an identical or higher/lower number of differentially spliced genes among its targets using the hypergeometric test, and corrected the results using Benjamini-Hochberg multiple testing correction. There were 296 RBP binding sites that were enriched for differentially spliced genes over all betacoronavirus datasets, compared to 686 in the other datasets (Figure 7A). Furthermore, there were 687 RBP binding sites whose target sets were depleted of differentially spliced genes in the betacoronavirus datasets, compared to 36 in the other datasets (Figure 7B).

### Differentially spliced genes of betacoronaviruses are enriched for protein complexes related to ribosome assembly

The CORUM database [51] contains data on experimentally characterized protein complexes. In order to obtain a better understanding of the role of differentially spliced genes in betacoronaviruses, we tested the set of CORUM core complexes for DAS gene enrichment using the hypergeometric test. Setting an FDR threshold of 0.05 using the Benjamini-Hochberg correction, *Nop56p-associated pre-rRNA complex* was enriched in 6 of the 8 betacoronavirus datasets that were downloaded from SRA.

Nop56p is a component of the box C/D small nucleolar ribonucleoprotein complexes that direct 2’-O-methylation of pre-rRNA during its maturation [52]. The Nop56p-associated pre-rRNA complex contains 61 ribosomal proteins including RPL10A, RPL5, RPS3A, RPL4, RPL3, RPL6, RPL22, which were shown to be differentially spliced in our study and are discussed below. Additionally, the CORUM protein complex *Ribosome, cytoplasmic* was enriched in 5 betacoronavirus datasets. Supplemental table S1 contains all the significant complex enrichments. DAS genes in datasets of other pathogens were not significantly enriched for complexes.

### Viral load is associated with isoform distribution of SARS-CoV-2 DAS genes

**Figure 8:**
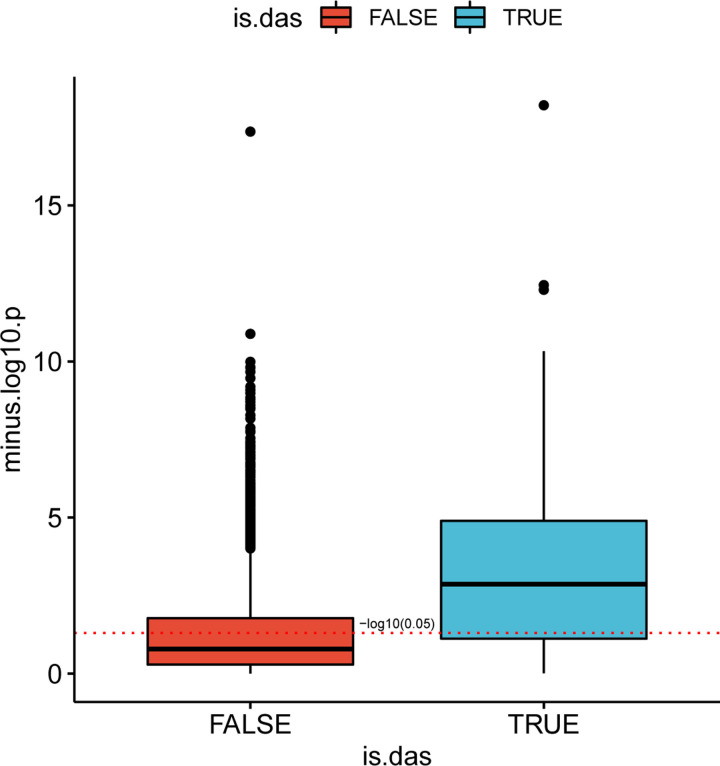
Isoform distribution and viral load. The correlation between viral load in each clinical sample and − log_10_(*p*) for isoforms that belong to DAS genes (blue) and for isoforms of non-DAS genes (red) is shown. For each isoform, the proportion of its counts out of the total number of isoform counts of its corresponding gene was calculated in each sample, and a Kendall correlation test between these values and the fractions of SARS-COV2 RNA was performed. The y-axis corresponds to the − log_10_ of the p-value. The red dashed line corresponds to a p-value of 0.05. The correlation between the raw proportions of differentially-spliced isoforms and suggest that the severity of viral infection as reflected in viral load may be a factor in determining the distribution of patterns of alternative splicing

We tested the correlation between the fraction of viral RNA and the proportion of counts of the different isoform of each differentially spliced gene in the SARS-COV-2-A dataset [27]. For each isoform fractions we performed a Kendall correlation test between the viral load and − log_10_(*p*) for isoforms that belong to DAS genes, and for isoforms of non-DAS genes. Isoforms of DAS genes are more highly correlated with viral load than isoforms of non-DAS genes (Fig. 8). An example for two isoforms of *ADAR* is shown in Supplemental Figure S4.

### Alternative splicing associated with SARS-CoV-2 affects a diverse set of genes and biological functions

It can be challenging to interpret the biological consequences of alternative splicing because experimental characterization of the biological functions of individual isoforms of most genes is not available. However, some of the alternative splicing events detected affected exons or isoforms with known or likely functional roles. Here we present selected alternative splicing events observed in the clinical SARS-CoV-2 nasal swab (NSPP) dataset. Detailed explanations, visualizations, and references are available in Supplemental Figures S5–S10.

**Figure 9:**
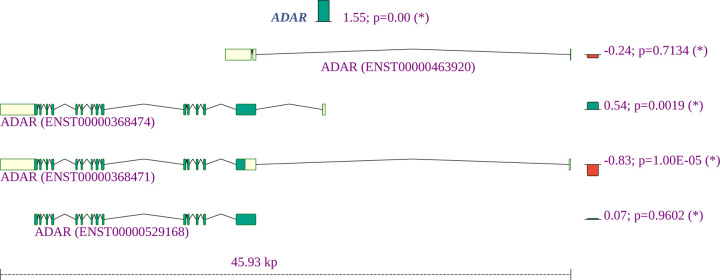
Summary of the gene expression and splicing profile of *ADAR* in COVID-19 patient samples. The expression of *ADAR* was increased by a factor of 2^1.55^ = 3.1. The proportion of isoforms containing two Z-DNA binding domains are increased, whereas an isoform expressing one such domain is decreased.

ADARs (adenosine deaminases that act on RNA) target double-stranded RNA (dsRNA) for deamination of adenines into inosines, and act during viral infections to produce hypermutation of the viral RNA or to edit host transcripts that modulate the cellular response. ADARs have been show to be involved in coronavirus genome editing [53]. We find that the overall expression of ADAR is increased in COVID-19 patient samples as compared to controls, and in addition, isoforms containing two Z-DNA binding domains are increased whereas an isoform expressing only one such domain is decreased. The shorter isoform with one Z-DNA binding domain is constitutively expressed. The longer isoform is expressed in response to interferon from a different promoter [54]. The smaller isoform is almost exclusively found in the nucleus while the larger is expressed in the cytoplasm [55] (Fig. 9).

Adenosine deamination in double-stranded RNAs is mediated by adenosine deaminase acting on RNA (ADAR) enzymes, which can convert adenosine to inosine residues. ADAR enzymes are thought to act on the SARS-CoV-2 genome [53, 56]. Differential splicing of ADAR is likely to affect overall activity of the enzyme and could contribute to the proposed role of ADAR in coronavirus genome editing.

The expression of *NCOA7* was significantly increased. The short isoform of *NCOA7* is inducible by interferon *β* and may play a role in resistance to inflammation-mediated oxidative stress [57]. In our study, the short isoform showed a tendency towards increased expression and the long isoform was significantly underexpressed.

*OAS2*, which was previously shown to be highly overexpressed in lung adenocarcinomal cells infected with SARS-CoV-2 [58], displayed a fold change of 7.11 in the NSPP samples. In addition, a short isoform was a substantially underexpressed compared to the long isoform. The three oligoadenylate synthetases play critical roles in cellular innate antiviral response [59]. The longer isoform of *OAS2* contains two oligoadenylate synthase domains, while the short form contains only one (Supplemental Fig. S6).

Strikingly, several ribosomal proteins show both reduced overall expression and a shift from coding to non-coding isoforms, including *RPL10A*, *RPL22*, *RPL3*,*RPL4*, *RPL5*, *RPL6*, *RPS3A*, and *RPS4X* (Supplemental Fig. S7 shows an example). The small subunit of the ribosome contains one 18S rRNA and about 32 ribosomal proteins (RPs) while, the large 60S subunit consists of 47 ribosomal proteins and one rRNA of 5S, 5.8S, and 28S. This suggests that altered alternative splicing of ribosomal proteins may contribute to the recently described disruption of translation attributed to SARS-CoV-2 infection [14]. In the NSPP dataset, 34 of 409 (8.3%) alternatively spliced genes are annotated to *protein targeting to ER* (GO:0045047), a proportion which is almost 4 times higher than in the population (67/3237; 2.1%). Many ribosomal proteins (RPs) interact with viral mRNA and proteins to participate in viral protein biosynthesis and regulate the replication and infection of virus in host cells [45].

A number of genes were found by HBA-DEALS analysis to be not differentially expressed but to show differential splicing, including *CLSTN1*, *G3BP1*, and *SMAD3*. *CLSTN1* encodes calsyntenin, which mediates transport of endosomes along microtubules in neurons as well as mediating intracellular transport of endosomes in HCV-infected cells, thereby contributing to the early stages of the viral replication cycle [60]. A long isoform of *CLSTN1* showed increased expression in the clinical samples (Supplemental Fig. S8). *G3BP1* encodes Ras GTPase-activating protein-binding protein 1, an ATP- and magnesium-dependent helicase that plays an essential role in innate immunity that was shown to play an antiviral role against porcine epidemic diarrhea virus, which is a single-stranded, positive-sense RNA virus that belongs to the Coronaviridae [61]. Alternative splicing in clinical samples is a shift to the shorter isoform, which lacks a RNA recognition motif (RRM) domain (Supplemental Fig. S9). *SMAD3* encodes an intracellular effector of gene expression responses to TGF-*β*, which can be transcriptionally induced following a number of different viral infections and may promote survival and growth of intracellular pathogens [62]. The SARS-associated coronavirus nucleocapsid protein interacts with Smad3 and modulates transforming growth factor-*β* signaling [63]. In the clinical samples, we noted a shift to a non-coding *SMAD3* isoform (Supplemental Fig. S10).

## Discussion

Our study has shown widespread differential alternative splicing associated with SARSCoV-2, SARS, and MERS infection, affecting genes involved with a characteristic set of functions. We characterized genes showing significant alternative splicing in clinical samples (nasal swabs) of patients with acute COVID-19. Our results provide a catalog of patterns of alternative splicing of potential relevance for understanding the biology of COVID-19 infection.

Although mechanisms differ from virus to virus, the general cycle of infection of a virus involves four major steps: (i) attachment to and entering into a host cell; (ii) replication and transcription of the viral genome followed by translation of viral mRNA; and (iii) assembly into progeny virions; (iv) release of virions from the infected cell. Viruses leverage cellular enzymes to implement these steps. Our analysis identified alternative splicing events potentially affecting each of these functions. For instance, 22 of 175 (12.6%) genes showing alternative splicing in lung tissue infected by SARS-CoV-2 were annotated to *cadherin binding* (GO:0045296), a proportion that is over three times higher than in the population of all genes with at least one read count (239/6435, or 3.7%). One of the differentially spliced genes is *EGFR*. It is known that many viruses usurp EGFR endocytosis or EGFR-mediated signalling for entry into the host cell and other purposes [64]. *IFI16* plays a role in *negative regulation of viral genome replication* (GO:0045071) [65].

**Figure 10:**
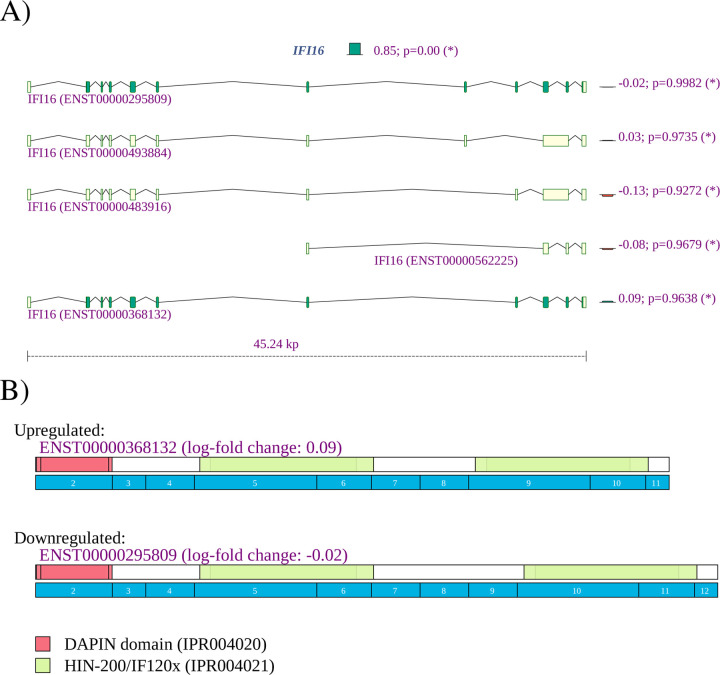
*IFI16* **A**) The expression of *IFI16* is increased with a fold change of 2^2.40^ = 5.23. Reads were mapped to two of the 14 isoforms noted in Ensembl. The expression of isoform ENST00000295809, corresponding to IFI16A, was 2^0.85^ = 1.80 higher in lung samples from COVID-19 patients, and the expression of isoform ENST00000448393, corresponding to IFI16C, was 2^−1.95^ or 3.86 times lower.

However, computational analysis of such datasets remains challenging because limited information is available about specific functions of individual isoforms. Our observation of differential splicing of *IFI16* in SARS-CoV-2 infected lung tissue (SRP279203) is a case in point. *IFI16* is a member of the interferon (IFN)-inducible p200-protein family, all of whose members share a partially-conserved repeat of 200-amino acid residues (also called HIN-200 domain, Prosite:PS50834) in the C-terminus. Additionally, most proteins also share a protein-protein interaction DAPIN domain (prosite:PS50824) in the N-terminus [66, 67]. The IFI16 protein can sense cytosolic as well as nuclear dsDNA and can initiate different innate immune responses. In previous literature, three isoforms of are described, with IFI16A containing all exons, IFI16B not including an exon termed 7a (exon 9 of ENST00000295809.12, exon id ENSE00003664980), and ) IFI16C not including exons ”7” (exon 8 of ENST00000295809.12, exon id ENSE00003481880) and 7a [68]. IFI16A can thus contain one, two, or three copies of highly conserved 56-amino acids serine-threonine-proline (S/T/P)-rich spacer region. IFI16 was shown to influence both glucocorticoid receptor (GR) transactivation and transrepression via an interaction that was specific to the B isoform [69]. The IFI16 B isoform has been reported to be selectively upregulated in the inflammatory disease systemic lupus erythematosus [70]. It is not always straightforward to use information like this to interpret findings from RNA-seq studies. Currently, a total of 14 *IFI16* isoforms are registered in Ensembl. We observed differential splicing for isoforms that correspond to isoform A and isoform C in the older literature (Figure 10). It is currently unknown whether isoforms with three (A) or one (C) copy of the spacer region have specific functionality. Our findings suggest that the cellular response to SARS-CoV-2 infection in the lung involves both up-regulation of *IFI16* as well as a shift from isoforms with one spacer region to isoforms with three copies. This, and many other similar findings illustrate both the limits of our knowledge of the biological roles of alternative splicing and highlight targets for hypothesis driven research on the functions of differentially spliced isoforms.

Our current data on ADAR must be viewed in the context of the complex, multi-tasking role that this protein plays in immune responses in viral infections and cancer and how the splicing variants we identify factor into these roles. ADAR forms can be important, through interactions with multiple other proteins for controlling levels and directions of immune changes [55, 71]. Key examples include that ADAR has been recently emphasized as important for causing immune suppression and the mechanisms are still being defined [55, 71]. In this regard, ADAR balances self-tolerance and immune activity by modulating canonical antiviral pathways induced by dsRNA [72]. Adenosine to inosine editing or binding of the cytoplasmic ADAR1 isoform p150 and/or the nuclear p110 to dsRNA prevents causes these species to escape cytoplasmic antiviral signaling pathways, via interactions with the RIG-I like receptor-, OAS/RNAseL-, and PKR pathways [55]. This role can be directed towards dsRNA species of viral origin, or, with reduced ADAR levels, endogenous dsRNAs including from inverted Alu repeats and particularly from dsRNA’s residing in mitochondria [55]. In fact, control of inflammasome signaling can be controlled in mitochondria by effects of ADAR on activity of a key protein CMPK2 (PKR). Prior studies have shown that ADAR1 can inhibit viral RNA mediated PKR activation [73] and more recently levels of ADAR have been shown to block the action of CMPK2 in accelerating inflammasome signaling [74]. Further, in viral infections, ADAR is involved in immune antiviral signaling, through regulation of IFN-I production and induction of cellular translation arrest, and apoptosis [73, 74]. These latter activities must be tightly regulated in order to not create an environment that favors virus replication. Finally, it has been proposed that ADAR plays a ‘dual’ protective role against autoinflammatory disease by regulating ‘IFN production’ and the response to IFN. This is especially apparent when ADAR is mutated in a form of childhood “interferonopathy” wherein there is resultant increase in MDA5-mediated ‘IFN production’ in specific cell types such as neuronal lineage, probably explaining resultant severe neuropathology [74].

In addition to providing a comprehensive atlas of genes showing differential splicing related to SARS-CoV-2, we have shown a striking overlap in the functional roles of genes displaying DAS in samples infected with any of the three betacoronaviruses SARS-CoV, SARS-CoV-2, and MERS. We have shown a higher proportion of intron-retention isoforms in cells infected by coronaviruses as compared to a control group of cells infected by *Streptococcus pneumonia*, HCV, Zika virus, Dengue virus, influenza H3N2, and RSV. Speculatively, this could be related to the global suppression of mRNA splicing thought to be due to NSP16 binding to the mRNA recognition domains of the U1 and U2 splicing RNAs [14]. We additionally showed that DAS genes identified in coronavirus-infected samples tend to have a lower number of exons than DAS genes in the control groups. We have no mechanistic explanation for this observation.

In summary, our study provides a comprehensive atlas of genes showing differential alternative splicing associated with infection by SARS-CoV-2, other betacoronaviruses, and a control set of unrelated viruses. Differential alternative splicing occurs in a diverse range of genes that perform a broad range of functions. We found characteristic enrichment of functions related to mRNA binding and splicing, gene expression, and endoplasmic reticulum, among other functions. Our study identified a number of associations that may provide hypotheses for future targeted studies, including increased intron retention, depletion of RBP binding sites in differentially spliced genes, and association of exons affected by alternative splicing with several RNA modifications, and an association of genes affected by alternative splicing with ribosomal complexes.

## Supplementary Material

Supplement 1

## Figures and Tables

**Table 1:** Summary of RNA-seq transcriptional profiling experiments. Data were taken from published experiments in which cell lines were inoculated with an infectious agent and compared to mock inoculations. Datasets were identified in the NCBI short-read archive. Columns: *Inf.-Mock*: number of infected/mock samples in an experiment. *ID*: identifier used in this work. Abbreviations: DENV: Dengue virus; ECs: epithelial cells; hNEC: human nasal epithelial cells; MERS: Middle East Respiratory Syndrome Coronavirus; PBMC: Peripheral blood mononuclear cells; SARS: Severe acute respiratory syndrome-associated coronavirus. Strep: *Streptococcus pneumoniae*. RSV: Respiratory Syncytial Virus. The three datasets from SRP040070 were the high multiplicity of infection (MOI) experiments chosen from among a larger set of experiments. NSP1 (NSP2): SARS-CoV-2 NSP1 (NSP2) transfection 24h.

Virus	Cell line /Tissue	SRP	Inf.-Mock	Citation	ID
SARS (24h)	MRC5	SRP040070	6 – 3	n/a	SARS
MERS (24h)	MRC5	SRP040070	3 – 3	n/a	MERS-24h
MERS (48h)	MRC5	SRP040070	3 – 3	n/a	MERS-48h
MERS (24h)	Calu-3	SRP227272	6 – 6	[21]	MERS-Calu3
Strep	Nasal ECs	SRP178454	5 – 8	[22]	Strep
HCV (day 1)	Huh7.5.1	SRP186406	3– 3	[23]	HCV
Zika	A549	SRP251704	3 – 3	[24]	Zika
DENV	A549	SRP078309	3 – 3	[9]	DENV
H3N2	hNEC	SRP222569	10 – 8	[25]	H3N2-NEC
H3N2	lung	SRP216763	5 – 5	[26]	H3N2-lung
RSV	Nasal Scrape	SRP273785	16 – 32	n/a	RSV
SARS-CoV-2 inf.	NPSS	-	100– 193	[27]	SARS-COV-2-A
SARS-CoV-2 inf.	lung	SRP279203	5 – 5	n/a	SARS-COV-2-B
SARS-CoV-2 inf.	Nasal ECs	SRP294125	3 – 3	[28]	SARS-COV-2-C
NSP1	HEK293	SRP284977	3 – 3	[29]	NSP1
NSP2	HEK293	SRP284977	3 – 3	[29]	NSP2

**Table 2:** Datasets. Summary of read mapping and HBA-DEALS analysis. Filtered: Mean number of filtered reads; Len: Mean read length; Unique: Mean Fraction Uniquely Mapped Reads; Unmapped: Mean Fraction Unmapped Reads. DGE: Number of differentially expressed genes; DAS: Number of differentially spliced genes. SARS-COV-2-A data were processed using the pipelines developed for COV-iRT [27]. Other datasets were processed as described in the methods.

Dataset ID	Filtered	Len	Unique	Unmapped	DGE	DAS
SARS	86660648	201	0.93	0.000312	1218	1441
MERS-24h	96741100	201	0.679	0.000246	2812	2656
MERS-48h	94307900	201	0.625	0.000305	7376	7394
MERS-Calu3	23245300	286.9	0.515	0.00159	6142	2141
Strep	47362500	149	0.774	0.000551	82	104
HCV	2926060	50	0.598	0.000634	35	113
Zika	48094200	299	0.924	0.00196	327	1103
DENV	35217400	197.7	0.902	0.00157	2496	1530
H3N2-NEC	10621100	250.9	0.894	0.0024	534	33
H3N2-lung	78239200	235.7	0.913	0.00269	190	110
RSV	48996100	101	0.861	0.000477	33	51
SARS-CoV-2-A	-	-	-	-	445	96
SARS-CoV-2-B	32746900	199.1	0.373	0.00135	96	174
SARS-CoV-2-C	47053600	281.5	0.638	0.0014	545	766
NSP1	33794100	99.5	0.12	0.00161	297	166
NSP2	36012300	99.5	0.123	0.0016	153	143

## References

[1] Yuki Koichi, Fujiogi Miho, and Koutsogiannaki Sophia. Covid-19 pathophysiology: A review. Clinical immunology (Orlando, Fla.), 215:108427, June 2020. 10.1016/j.clim.2020.108427 PMC7169933 32325252

[2] Puelles V. G., Lütgehetmann M., Lindenmeyer M. T., Sperhake J. P., Wong M. N., Allweiss L., Chilla S., Heinemann A., Wanner N., Liu S., Braun F., Lu S., Pfefferle S., Schröder A. S., Edler C., Gross O., Glatzel M., Wichmann D., Wiech T., Kluge S., Pueschel K., Aepfelbacher M., and Huber T. B.. Multiorgan and Renal Tropism of SARS-CoV-2. N Engl J Med, 383(6):590–592, 08 2020. 32402155 10.1056/NEJMc2011400 PMC7240771

[3] Wiersinga W Joost, Rhodes Andrew, Cheng Allen C, Peacock Sharon J, and Prescott Hallie C. Pathophysiology, transmission, diagnosis, and treatment of coronavirus disease 2019 (covid-19): A review. JAMA, 324:782–793, August 2020. 32648899 10.1001/jama.2020.12839

[4] Baralle Francisco E. and Giudice Jimena. Alternative splicing as a regulator of development and tissue identity. Nature reviews. Molecular cell biology, 18:437–451, July 2017. 28488700 10.1038/nrm.2017.27 PMC6839889

[5] Nilsen Timothy W. and Graveley Brenton R.. Expansion of the eukaryotic proteome by alternative splicing. Nature, 463:457–463, January 2010. 20110989 10.1038/nature08909 PMC3443858

[6] Lad Sonya P., Yang Guang, Scott David A., Chao Ta-Hsiang, da Silva Correia Jean, de la Torre Juan Carlos, and Li Erguang. Identification of mavs splicing variants that interfere with rigi/mavs pathway signaling. Molecular immunology, 45:2277–2287, April 2008. 18207245 10.1016/j.molimm.2007.11.018

[7] Gack Michaela U., Kirchhofer Axel, Shin Young C., Inn Kyung-Soo, Liang Chengyu, Cui Sheng, Myong Sua, Ha Taekjip, Hopfner Karl-Peter, and Jung Jae U.. Roles of RIG-I N-terminal tandem CARD and splice variant in trim25-mediated antiviral signal transduction. Proceedings of the National Academy of Sciences of the United States of America, 105:16743–16748, October 2008. 18948594 10.1073/pnas.0804947105 PMC2575490

[8] Brubaker Sky W., Gauthier Anna E., Mills Eric W., Ingolia Nicholas T., and Kagan Jonathan C.. A bicistronic MAVS transcript highlights a class of truncated variants in antiviral immunity. Cell, 156:800–811, February 2014. 24529381 10.1016/j.cell.2014.01.021 PMC3959641

[9] De Maio Federico A., Risso Guillermo, Iglesias Nestor G., Shah Priya, Pozzi Berta, Gebhard Leopoldo G., Mammi Pablo, Mancini Estefania, Yanovsky Marcelo J., Andino Raul, Krogan Nevan, Srebrow Anabella, and Gamarnik Andrea V.. The Dengue virus NS5 protein intrudes in the cellular spliceosome and modulates splicing. PLoS pathogens, 12:e1005841, August 2016. 27575636 10.1371/journal.ppat.1005841 PMC5004807

[10] Koop Anja, Lepenies Inga, Braum Oliver, Davarnia Parvin, Scherer Gudrun, Fickenscher Helmut, Kabelitz Dieter, and Adam-Klages Sabine. Novel splice variants of human ikkϵ negatively regulate ikkϵ-induced irf3 and nf-kb activation. European journal of immunology, 41:224–234, January 2011. 21182093 10.1002/eji.201040814

[11] Ku Chia-Chi, Che Xi-Bing, Reichelt Mike, Rajamani Jaya, Schaap-Nutt Anne, Huang Ke-Jung, Sommer Marvin H., Chen Yi-Shun, Chen Yi-Yuan, and Arvin Ann M.. Herpes simplex virus-1 induces expression of a novel mxa isoform that enhances viral replication. Immunology and cell biology, 89:173–182, February 2011. 20603636 10.1038/icb.2010.83 PMC5520797

[12] Almstead Laura L. and Sarnow Peter. Inhibition of u snrnp assembly by a virus-encoded proteinase. Genes & development, 21:1086–1097, May 2007. 17473171 10.1101/gad.1535607 PMC1855234

[13] Zhang Liang, Wang Juan, Muñoz-Moreno Raquel, Kim Min, Sakthivel Ramanavelan, Mo Wei, Shao Dandan, Anantharaman Aparna, García-Sastre Adolfo, Conrad Nicholas K., and Fontoura Beatriz M. A.. Influenza virus ns1 protein-rna interactome reveals intron targeting. Journal of virology, 92, December 2018. 10.1128/JVI.01634-18 PMC6258958 30258002

[14] Banerjee Abhik K., Blanco Mario R., Bruce Emily A., Honson Drew D., Chen Linlin M., Chow Amy, Bhat Prashant, Ollikainen Noah, Quinodoz Sofia A., Loney Colin, Thai Jasmine, Miller Zachary D., Lin Aaron E., Schmidt Madaline M., Stewart Douglas G., Goldfarb Daniel, De Lorenzo Giuditta, Rihn Suzannah J., Voorhees Rebecca M., Botten Jason W., Majumdar Devdoot, and Guttman Mitchell. SARS-CoV-2 disrupts splicing, translation, and protein trafficking to suppress host defenses. Cell, 183:1325–1339.e21, November 2020. 33080218 10.1016/j.cell.2020.10.004 PMC7543886

[15] Decroly Etienne, Debarnot Claire, Ferron François, Bouvet Mickael, Coutard Bruno, Imbert Isabelle, Gluais Laure, Papageorgiou Nicolas, Sharff Andrew, Bricogne Gérard, Ortiz-Lombardia Miguel, Lescar Julien, and Canard Bruno. Crystal structure and functional analysis of the SARS-coronavirus rna cap 2’-o-methyltransferase nsp10/nsp16 complex. PLoS pathogens, 7:e1002059, May 2011. 21637813 10.1371/journal.ppat.1002059 PMC3102710

[16] Nakagawa Keisuke and Makino Shinji. Mechanisms of coronavirus nsp1-mediated control of host and viral gene expression. Cells, 10(2):300, February 2021. 33540583 10.3390/cells10020300 PMC7912902

[17] Lapointe CP, Grosely R, Johnson AG, Wang J, Fernández IS, and Puglisi JD. Dynamic competition between sars-cov-2 nsp1 and mrna on the human ribosome inhibits translation initiation. Proc Natl Acad Sci U S A., 118(6):e2017715118, 2021. 33479166 10.1073/pnas.2017715118 PMC8017934

[18] Zhang K, Miorin L, Makio T, Dehghan I, Gao S, Xie Y, Zhong H, Esparza M, Kehrer T, Kumar A, Hobman TC, Ptak C, Gao B, Minna JD, Chen Z, García-Sastre A, Ren Y, Wozniak RW, and Fontoura BMA. Nsp1 protein of sars-cov-2 disrupts the mrna export machinery to inhibit host gene expression. Sci Adv, 7(6):eabe7386, 2021. 33547084 10.1126/sciadv.abe7386 PMC7864571

[19] Leinonen Rasko, Sugawara Hideaki, Shumway Martin, and International Nucleotide Sequence Database Collaboration. The sequence read archive. Nucleic acids research, 39:D19–D21, January 2011. 21062823 10.1093/nar/gkq1019 PMC3013647

[20] Rodriguez-Frandsen Ariel, Martin-Sancho Laura, Gounder Anshu P, Chang Max W, Liu Wen-Chun, De Jesus Paul D, von Recum-Knepper Jessica, Dutra Miriam S, Huffmaster Nicholas J, Chavarria Monica, Mena Ignacio, Riva Laura, Nguyen Courtney B, Dobariya Saunil, Herbert Kristina M, Benner Christopher, Albrecht Randy A, García-Sastre Adolfo, and Chanda Sumit K. Viral determinants in H5N1 influenza A virus enable productive infection of HeLa cells. Journal of virology, 94, January 2020. 10.1128/JVI.01410-19 PMC6997754 31776276

[21] Zhang Xi, Chu Hin, Wen Lei, Shuai Huiping, Yang Dong, Wang Yixin, Hou Yuxin, Zhu Zheng, Yuan Shuofeng, Yin Feifei, Fuk-Woo Chan Jasper, and Yuen Kwok-Yung. Competing endogenous rna network profiling reveals novel host dependency factors required for mers-cov propagation. Emerging microbes & infections, 9:733–746, December 2020. 32223537 10.1080/22221751.2020.1738277 PMC7170352

[22] Weight Caroline M., Venturini Cristina, Pojar Sherin, Jochems Simon P., Reiné Jesús, Nikolaou Elissavet, Solórzano Carla, Noursadeghi Mahdad, Brown Jeremy S., Ferreira Daniela M., and Heyderman Robert S.. Microinvasion by streptococcus pneumoniae induces epithelial innate immunity during colonisation at the human mucosal surface. Nature communications, 10:3060, July 2019. 10.1038/s41467-019-11005-2 PMC6635362 31311921

[23] Lupberger Joachim, Croonenborghs Tom, Roca Suarez Armando Andres, Van Renne Nicolaas, Jühling Frank, Oudot Marine A., Virzì Alessia, Bandiera Simonetta, Jamey Carole, Meszaros Gergö, Brumaru Daniel, Mukherji Atish, Du-rand Sarah C., Heydmann Laura, Verrier Eloi R., El Saghire Hussein, Hamdane Nourdine, Bartenschlager Ralf, Fereshetian Shaunt, Ramberger Evelyn, Sinha Rileen, Nabian Mohsen, Everaert Celine, Jovanovic Marko, Mertins Philipp, Carr Steven A., Chayama Kazuaki, Dali-Youcef Nassim, Ricci Romeo, Bardeesy Nabeel M., Fujiwara Naoto, Gevaert Olivier, Zeisel Mirjam B., Hoshida Yujin, Pochet Nathalie, and Baumert Thomas F.. Combined analysis of metabolomes, proteomes, and transcriptomes of hepatitis c virus-infected cells and liver to identify pathways associated with disease development. Gastroenterology, 157:537–551.e9, August 2019. 30978357 10.1053/j.gastro.2019.04.003 PMC8318381

[24] Liao Xinzhong, Xie He, Li Shilin, Ye Haiyan, Li Shuang, Ren Kai, Li Yujia, Xu Min, Lin Wenyu, Duan Xiaoqiong, Yang Chunhui, and Chen Limin. 2’, 5’-oligoadenylate synthetase 2 (OAS2) inhibits Zika virus replication through activation of type I IFN signaling pathway. Viruses, 12, April 2020. 10.3390/v12040418 PMC7232345 32276512

[25] Tan Kai Sen, Andiappan Anand Kumar, Lee Bernett, Yan Yan, Liu Jing, Tang See Aik, Lum Josephine, He Ting Ting, Ong Yew Kwang, Thong Mark, Lim Hui Fang, Choi Hyung Won, Rotzschke Olaf, Chow Vincent T., and Wang De Yun. Rna sequencing of h3n2 influenza virus-infected human nasal epithelial cells from multiple subjects reveals molecular pathways associated with tissue injury and complications. Cells, 8, August 2019. 10.3390/cells8090986 PMC6770044 31461941

[26] da Rocha Matos Aline, Wunderlich Katharina, Schloer Sebastian, Schughart Klaus, Geffers Robert, Seders Martine, de Witt Marlous, Christersson Anmari, Wiewrodt Rainer, Wiebe Karsten, Barth Peter, Hocke Andreas, Hippenstiel Stefan, Hönzke Katja, Dittmer Ulf, Sutter Kathrin, Rescher Ursula, Rodionycheva Svetlana, Matera Nicoletta, Ludwig Stephan, and Brunotte Linda. Antiviral potential of human ifn-α subtypes against influenza a h3n2 infection in human lung explants reveals subtypespecific activities. Emerging microbes & infections, 8:1763–1776, 2019. 31826721 10.1080/22221751.2019.1698271 PMC6913622

[27] Butler D., Mozsary C., Meydan C., Foox J., Rosiene J., Shaiber A., Danko D., Afshinnekoo E., MacKay M., Sedlazeck F. J., Ivanov N. A., Sierra M., Pohle D., Zietz M., Gisladottir U., Ramlall V., Sholle E. T., Schenck E. J., Westover C. D., Hassan C., Ryon K., Young B., Bhattacharya C., Ng D. L., Granados A. C., Santos Y. A., Servellita V., Federman S., Ruggiero P., Fungtammasan A., Chin C. S., Pearson N. M., Langhorst B. W., Tanner N. A., Kim Y., Reeves J. W., Hether T. D., Warren S. E., Bailey M., Gawrys J., Meleshko D., Xu D., Couto-Rodriguez M., Nagy-Szakal D., Barrows J., Wells H., O’Hara N. B., Rosenfeld J. A., Chen Y., Steel P. A. D., Shemesh A. J., Xiang J., Thierry-Mieg J., Thierry-Mieg D., Iftner A., Bezdan D., Sanchez E., Campion T. R., Sipley J., Cong L., Craney A., Velu P., Melnick A. M., Shapira S., Hajirasouliha I., Borczuk A., Iftner T., Salvatore M., Loda M., West-blade L. F., Cushing M., Wu S., Levy S., Chiu C., Schwartz R. E., Tatonetti N., Rennert H., Imielinski M., and Mason C. E.. Shotgun transcriptome, spatial omics, and isothermal profiling of SARS-CoV-2 infection reveals unique host responses, viral diversification, and drug interactions. Nat Commun, 12(1):1660, 03 2021. 33712587 10.1038/s41467-021-21361-7 PMC7954844

[28] Gamage Akshamal M., Tan Kai Sen, Chan Wharton O. Y., Liu Jing, Tan Chee Wah, Ong Yew Kwang, Thong Mark, Andiappan Anand K., Anderson Danielle E., Wang De Yun, and Wang Lin-Fa. Infection of human nasal epithelial cells with SARS-CoV-2 and a 382-nt deletion isolate lacking ORF8 reveals similar viral kinetics and host transcriptional profiles. PLOS Pathogens, 16(12):e1009130, December 2020. 33284849 10.1371/journal.ppat.1009130 PMC7746279

[29] Rao Shilpa, Hoskins Ian, Garcia P. Daniela, Tonn Tori, Ozadam Hakan, Cenik Elif Sarinay, and Cenik Can. Genes with 5’ terminal oligopyrimidine tracts preferentially escape global suppression of translation by the sars-cov-2 nsp1 protein. bioRxiv : the preprint server for biology, September 2020. 10.1261/rna.078661.120 PMC8370740 34127534

[30] Chen Jing, Gálvez-Peralta Marina, Zhang Xiang, Deng Jingyuan, Liu Zijuan, and Nebert Daniel W. In utero gene expression in the slc39a8(neo/neo) knockdown mouse. Scientific reports, 8:10703, July 2018. 30013175 10.1038/s41598-018-29109-y PMC6048144

[31] Dobin Alexander, Davis Carrie A, Schlesinger Felix, Drenkow Jorg, Zaleski Chris, Jha Sonali, Batut Philippe, Chaisson Mark, and Gingeras Thomas R. STAR: ultrafast universal RNA-seq aligner. Bioinformatics (Oxford, England), 29:15–21, January 2013. 10.1093/bioinformatics/bts635 PMC3530905 23104886

[32] Li Bo and Dewey Colin N.. Rsem: accurate transcript quantification from rna-seq data with or without a reference genome. BMC bioinformatics, 12:323, August 2011. 21816040 10.1186/1471-2105-12-323 PMC3163565

[33] Wood Derrick E., Lu Jennifer, and Langmead Ben. Improved metagenomic analysis with kraken 2. Genome biology, 20:257, November 2019. 31779668 10.1186/s13059-019-1891-0 PMC6883579

[34] Bolger Anthony M., Lohse Marc, and Usadel Bjoern. Trimmomatic: a flexible trimmer for Illumina sequence data. Bioinformatics (Oxford, England), 30:2114–2120, August 2014. 10.1093/bioinformatics/btu170 PMC4103590 24695404

[35] Ewels Philip, Magnusson Måns, Lundin Sverker, and Käller Max. Multiqc: summarize analysis results for multiple tools and samples in a single report. Bioinformatics (Oxford, England), 32:3047–3048, October 2016. 10.1093/bioinformatics/btw354 PMC5039924 27312411

[36] Karlebach Guy, Hansen Peter, Ft Veiga Diogo, Steinhaus Robin, Danis Daniel, Li Sheng, Anczukow Olga, and Robinson Peter N.. Hba-deals: accurate and simultaneous identification of differential expression and splicing using hierarchical bayesian analysis. Genome biology, 21:171, July 2020. 32660516 10.1186/s13059-020-02072-6 PMC7358203

[37] Scott James G. and Berger James O.. An exploration of aspects of Bayesian multiple testing. Journal of Statistical Planning and Inference, 136(7):2144–2162, July 2006.

[38] Kruschke John K.. Rejecting or accepting parameter values in bayesian estimation. Advances in Methods and Practices in Psychological Science, 1(2):270–280, 2018.

[39] Bauer Sebastian, Grossmann Steffen, Vingron Martin, and Robinson Peter N.. Ontologizer 2.0–a multifunctional tool for GO term enrichment analysis and data exploration. Bioinformatics (Oxford, England), 24:1650–1651, July 2008. 10.1093/bioinformatics/btn250 18511468

[40] Smedley Damian, Haider Syed, Durinck Steffen, Pandini Luca, Provero Paolo, Allen James, Arnaiz Olivier, Hamza Awedh Mohammad, Baldock Richard, Barbiera Giulia, Bardou Philippe, Beck Tim, Blake Andrew, Bonierbale Merideth, Brookes Anthony J., Bucci Gabriele, Buetti Iwan, Burge Sarah, Cabau Cédric, Carlson Joseph W., Chelala Claude, Chrysostomou Charalambos, Cittaro Davide, Collin Olivier, Cordova Raul, Cutts Rosalind J., Dassi Erik, Di Genova Alex, Djari Anis, Esposito Anthony, Estrella Heather, Eyras Eduardo, Fernandez-Banet Julio, Forbes Simon, Free Robert C., Fujisawa Takatomo, Gadaleta Emanuela, Garcia-Manteiga Jose M., Goodstein David, Gray Kristian, Guerra-Assunção José Afonso, Haggarty Bernard, Han Dong-Jin, Han Byung Woo, Harris Todd, Harshbarger Jayson, Hastings Robert K., Hayes Richard D., Hoede Claire, Hu Shen, Hu Zhi-Liang, Hutchins Lucie, Kan Zhengyan, Kawaji Hideya, Keliet Aminah, Kerhornou Arnaud, Kim Sunghoon, Kinsella Rhoda, Klopp Christophe, Kong Lei, Lawson Daniel, Lazarevic Dejan, Lee Ji-Hyun, Letellier Thomas, Li Chuan-Yun, Lio Pietro, Liu Chu-Jun, Luo Jie, Maass Alejandro, Mariette Jerome, Maurel Thomas, Merella Stefania, Mohamed Azza Mostafa, Moreews Francois, Nabihoudine Ibounyamine, Ndegwa Nelson, Noirot Céline, Perez-Llamas Cristian, Primig Michael, Quattrone Alessandro, Quesneville Hadi, Rambaldi Davide, Reecy James, Riba Michela, Rosanoff Steven, Saddiq Amna Ali, Salas Elisa, Sallou Olivier, Shepherd Rebecca, Simon Reinhard, Sperling Linda, Spooner William, Staines Daniel M., Steinbach Delphine, Stone Kevin, Stupka Elia, Teague Jon W., Dayem Ullah Abu Z., Wang Jun, Ware Doreen, Wong-Erasmus Marie, Youens-Clark Ken, Zadissa Amonida, Zhang Shi-Jian, and Kasprzyk Arek. The biomart community portal: an innovative alternative to large, centralized data repositories. Nucleic acids research, 43:W589–W598, July 2015. 25897122 10.1093/nar/gkv350 PMC4489294

[41] Meyer F.. Viral interactions with components of the splicing machinery. Progress in molecular biology and translational science, 142:241–268, 2016. 27571697 10.1016/bs.pmbts.2016.05.008

[42] Boudreault Simon, Roy Patricia, Lemay Guy, and Bisaillon Martin. Viral modulation of cellular RNA alternative splicing: A new key player in virus-host interactions? Wiley interdisciplinary reviews. RNA, 10:e1543, September 2019. 31034770 10.1002/wrna.1543 PMC6767064

[43] Chauhan Komal, Kalam Haroon, Dutt Ravi, and Kumar Dhiraj. RNA splicing: A new paradigm in host-pathogen interactions. Journal of molecular biology, 431:1565–1575, April 2019. 30857970 10.1016/j.jmb.2019.03.001 PMC7115970

[44] Choi Ji-Ae and Song Chang-Hwa. Insights into the role of endoplasmic reticulum stress in infectious diseases. Frontiers in immunology, 10:3147, 2019. 32082307 10.3389/fimmu.2019.03147 PMC7005066

[45] Li Shuo. Regulation of ribosomal proteins on viral infection. Cells, 8, May 2019. 10.3390/cells8050508 PMC6562653 31137833

[46] Walsh Derek, Mathews Michael B., and Mohr Ian. Tinkering with translation: protein synthesis in virus-infected cells. Cold Spring Harbor perspectives in biology, 5:a012351, January 2013. 23209131 10.1101/cshperspect.a012351 PMC3579402

[47] Schubert Katharina, Karousis Evangelos D., Jomaa Ahmad, Scaiola Alain, Echeverria Blanca, Gurzeler Lukas-Adrian, Leibundgut Marc, Thiel Volker, Oliver Mühlemann, and Nenad Ban. SARS-CoV-2 nsp1 binds the ribosomal mRNA channel to inhibit translation. Nature Structural & Molecular Biology, 27(10):959–966, September 2020. 10.1038/s41594-020-0511-8 32908316

[48] Karijolich John, Yi Chengqi, and Yu Yi-Tao. Transcriptome-wide dynamics of RNA pseudouridylation. Nature Reviews Molecular Cell Biology, 16(10):581–585, August 2015. 26285676 10.1038/nrm4040 PMC5694666

[49] Xuan Jia-Jia, Sun Wen-Ju, Lin Peng-Hui, Zhou Ke-Ren, Liu Shun, Zheng Ling-Ling, Qu Liang-Hu, and Yang Jian-Hua. RMBase v2.0: deciphering the map of RNA modifications from epitranscriptome sequencing data. Nucleic Acids Research, 46(D1):D327–D334, October 2017. 10.1093/nar/gkx934 PMC5753293 29040692

[50] Benoit Bouvrette Louis Philip, Bovaird Samantha, Blanchette Mathieu, and Lécuyer Eric. oRNAment: a database of putative RNA binding protein target sites in the transcriptomes of model species. Nucleic Acids Research, November 2019. 10.1093/nar/gkz986 PMC7145663 31724725

[51] Giurgiu Madalina, Reinhard Julian, Brauner Barbara, Dunger-Kaltenbach Irmtraud, Fobo Gisela, Frishman Goar, Montrone Corinna, and Ruepp Andreas. CORUM: the comprehensive resource of mammalian protein complexes—2019. Nucleic Acids Research, 47(D1):D559–D563, October 2018. 10.1093/nar/gky973 PMC6323970 30357367

[52] Hayano T., Yanagida M., Yamauchi Y., Shinkawa T., Isobe T., and Takahashi N.. Proteomic analysis of human Nop56p-associated pre-ribosomal ribonucleoprotein complexes. Possible link between Nop56p and the nucleolar protein treacle responsible for Treacher Collins syndrome. J Biol Chem, 278(36):34309–34319, Sep 2003. 12777385 10.1074/jbc.M304304200

[53] Di Giorgio Salvatore, Martignano Filippo, Torcia Maria Gabriella, Mattiuz Giorgio, and Conticello Silvestro G.. Evidence for host-dependent rna editing in the transcriptome of sars-cov-2. Science advances, 6:eabb5813, June 2020. 32596474 10.1126/sciadv.abb5813 PMC7299625

[54] George C. X. and Samuel C. E.. Human RNA-specific adenosine deaminase ADAR1 transcripts possess alternative exon 1 structures that initiate from different promoters, one constitutively active and the other interferon inducible. Proceedings of the National Academy of Sciences of the United States of America, 96:4621–4626, April 1999. 10200312 10.1073/pnas.96.8.4621 PMC16382

[55] Lamers Mart M., van den Hoogen Bernadette G., and Haagmans Bart L.. Adar1: ”editor-in-chief” of cytoplasmic innate immunity. Frontiers in immunology, 10:1763, 2019. 31404141 10.3389/fimmu.2019.01763 PMC6669771

[56] Mourier T., Sadykov M., Carr M. J., Gonzalez G., Hall W. W., and Pain A.. Host-directed editing of the SARS-CoV-2 genome. Biochem Biophys Res Commun, 538:35–39, 01 2021. 33234239 10.1016/j.bbrc.2020.10.092 PMC7643664

[57] Yu Lijian, Croze Ed, Yamaguchi Ken D., Tran Tiffany, Reder Anthony T., Litvak Vladimir, and Volkert Michael R.. Induction of a unique isoform of the ncoa7 oxidation resistance gene by interferon *β*−1b. Journal of interferon & cytokine research : the official journal of the International Society for Interferon and Cytokine Research, 35:186–199, March 2015. 10.1089/jir.2014.0115 PMC4350146 25330068

[58] Soremekun Opeyemi S., Omolabi Kehinde F., and Soliman Mahmoud E. S.. Identification and classification of differentially expressed genes reveal potential molecular signature associated with sars-cov-2 infection in lung adenocarcinomal cells. Informatics in medicine unlocked, 20:100384, 2020. 32835074 10.1016/j.imu.2020.100384 PMC7308782

[59] Sarkar S. N., Ghosh A., Wang H. W., Sung S. S., and Sen G. C.. The nature of the catalytic domain of 2’−5’-oligoadenylate synthetases. The Journal of biological chemistry, 274:25535–25542, September 1999. 10464285 10.1074/jbc.274.36.25535

[60] Awan Zunaira, Tay Enoch S. E., Eyre Nicholas S., Wu Lindsay E., Beard Michael R., Boo Irene, Drummer Heidi E., George Jacob, and Douglas Mark W.. Calsyntenin-1 mediates hepatitis c virus replication. The Journal of general virology, 97:1877–1887, August 2016. 27221318 10.1099/jgv.0.000511

[61] Pandey Kabita, Zhong Shuhong, Diel Diego G., Hou Yixuan, Wang Qiuhong, Nelson Eric, and Wang Xiuqing. Gtpase-activating protein-binding protein 1 (g3bp1) plays an antiviral role against porcine epidemic diarrhea virus. Veterinary microbiology, 236:108392, September 2019. 31500725 10.1016/j.vetmic.2019.108392 PMC7117524

[62] Qing Jing, Liu Cheng, Choy Lisa, Wu Rui-Yun, Pagano Joseph S., and Derynck Rik. Transforming growth factor beta/smad3 signaling regulates irf-7 function and transcriptional activation of the beta interferon promoter. Molecular and cellular biology, 24:1411–1425, February 2004. 14729983 10.1128/MCB.24.3.1411-1425.2004 PMC321430

[63] Zhao Xingang, Nicholls John M., and Chen Ye-Guang. Severe acute respiratory syndrome-associated coronavirus nucleocapsid protein interacts with smad3 and modulates transforming growth factor-beta signaling. The Journal of biological chemistry, 283:3272–3280, February 2008. 18055455 10.1074/jbc.M708033200 PMC8740907

[64] Zheng Kai, Kitazato Kaio, and Wang Yifei. Viruses exploit the function of epidermal growth factor receptor. Reviews in medical virology, 24:274–286, July 2014. 24888553 10.1002/rmv.1796

[65] Gariano Grazia Rosaria, Dell’Oste Valentina, Bronzini Matteo, Gatti Deborah, Luganini Anna, De Andrea Marco, Gribaudo Giorgio, Gariglio Marisa, and Landolfo Santo. The intracellular DNA sensor IFI16 gene acts as restriction factor for human cytomegalovirus replication. PLoS pathogens, 8:e1002498, January 2012. 22291595 10.1371/journal.ppat.1002498 PMC3266931

[66] Asefa Benyam, Klarmann Kimberly D., Copeland Neal G., Gilbert Debra J., Jenkins Nancy A., and Keller Jonathan R.. The interferon-inducible p200 family of proteins: a perspective on their roles in cell cycle regulation and differentiation. Blood cells, molecules & diseases, 32:155–167, 2004. 10.1016/j.bcmd.2003.10.002 14757431

[67] Veeranki Sudhakar and Choubey Divaker. Interferon-inducible p200-family protein ifi16, an innate immune sensor for cytosolic and nuclear double-stranded dna: regulation of subcellular localization. Molecular immunology, 49:567–571, January 2012. 22137500 10.1016/j.molimm.2011.11.004 PMC3249514

[68] Johnstone R. W., Kershaw M. H., and Trapani J. A.. Isotypic variants of the interferon-inducible transcriptional repressor IFI 16 arise through differential mRNA splicing. Biochemistry, 37:11924–11931, August 1998. 9718316 10.1021/bi981069a

[69] Berry A., Matthews L., Jangani M., Plumb J., Farrow S., Buchan N., Wilson P. A., Singh D., Ray D. W., and Donn R. P.. Interferon-inducible factor 16 is a novel modulator of glucocorticoid action. FASEB journal : official publication of the Federation of American Societies for Experimental Biology, 24:1700–1713, June 2010. 20086048 10.1096/fj.09-139998 PMC3000051

[70] Kimkong I., Avihingsanon Y., and Hirankarn N.. Expression profile of hin200 in leukocytes and renal biopsy of sle patients by real-time rt-pcr. Lupus, 18:1066–1072, October 2009. 19762380 10.1177/0961203309106699

[71] Herbert Alan. Adar and immune silencing in cancer. Trends in cancer, 5:272–282, May 2019. 31174840 10.1016/j.trecan.2019.03.004

[72] Jacki E. Heraud-Farlow and Carl R. Walkley. The role of rna editing by adar1 in prevention of innate immune sensing of self-rna. Journal of molecular medicine (Berlin, Germany), 94:1095–1102, October 2016. 10.1007/s00109-016-1416-1 27044320

[73] Jean-François Gélinas Guerline Clerzius, Shaw Eileen, and Gatignol Anne. Enhancement of replication of rna viruses by adar1 via rna editing and inhibition of rna-activated protein kinase. Journal of virology, 85:8460–8466, September 2011. 21490091 10.1128/JVI.00240-11 PMC3165853

[74] Chung Hachung, Calis Jorg J. A., Wu Xianfang, Sun Tony, Yu Yingpu, Sarbanes Stephanie L., Dao Thi Viet Loan, Shilvock Abigail R., Hoffmann H.-Heinrich, Rosenberg Brad R., and Rice Charles M.. Human adar1 prevents endogenous rna from triggering translational shutdown. Cell, 172:811–824.e14, February 2018. 29395325 10.1016/j.cell.2017.12.038 PMC5831367

